# Community narratives about women and HIV risk in 21 high-burden communities in Zambia and South Africa

**DOI:** 10.2147/IJWH.S143397

**Published:** 2017-11-29

**Authors:** Lario Viljoen, Rhoda Ndubani, Virginia Bond, Janet Seeley, Lindsey Reynolds, Graeme Hoddinott

**Affiliations:** 1 Desmond Tutu TB Centre, Department of Paediatrics and Child Health, Faculty of Medicine and Health Sciences, Stellenbosch University, Cape Town, South Africa; 2 Zambia AIDS-related Tuberculosis Project (Zambart), School of Medicine, Lusaka, Zambia; 3 Department of Global Health and Development, Faculty of Public Health and Policy, London School of Hygiene and Tropical Medicine, London, UK; 4 Population Studies and Training Center, Brown University, Providence, RI, USA; 5 Department of Sociology and Social Anthropology, Stellenbosch University, Stellenbosch, South Africa

**Keywords:** HIV/AIDS, gender, vulnerability, community beliefs

## Abstract

Public health researchers repeatedly represent women as a group vulnerable to ill health. This has been particularly true in the field of HIV research, where women are disproportionately affected by HIV in terms of disease burden and the social effects of the epidemic. Although women have been the focus of many prevention and treatment programs, structural barriers to implementation of these targeted programs persist. In this article we explore how high HIV-burden communities in South Africa and Zambia engage with the concepts of “woman” and “HIV risk”. The data are drawn from participatory storytelling activities completed with 604 participants across 78 group discussions between December 2012 and May 2013. During discussions we found that participants made use of the core archetypal caricatures of “goodness,” “badness,” and “vulnerability” when describing women’s HIV risk. Community members shifted between these categories in their characterizations of women, as they acknowledged the multiple roles women play, internalized different stories about women, and sometimes shifted register in the same stories. Findings suggest that health implementers, in consultation with community members, should consider the multiple positions women occupy and how this impacts the wider community’s understandings of women and “risk”. This approach of taking on board community understandings of the complexity of HIV risk can inform the design and implementation of HIV prevention and care programs by rendering programs more focused and in-line with community needs.

## Introduction

In sub-Saharan Africa, the epidemiological distribution of HIV is distinctly gendered. In South Africa, 12.2% of the population is HIV positive, which includes 14.4% of women compared to 9.9% of men.^1^ It is estimated that adult women (aged ≥15) account for >3.8 million of the 7 million people living with HIV (PLHIV) in South Africa.^2^ In Zambia, where HIV prevalence among adults aged 15–49 is 13%, 15% of all women and 11% of all men are HIV positive.^3^ Of the 1.1 million adults affected by HIV in Zambia, ~640,000 (58%) are women.^2^ The skewed distribution of HIV between women and men has amplified a long-standing narrative in public health and medicine, and in popular culture that women are a “vulnerable” group, in need of sympathy or support.^4^ Social constructions of vulnerability are grounded in part in the disproportionate biological risk, and the epidemiological and social burdens experienced by women.^5^–^7^ The physiological composition of women’s bodies makes women biologically more likely to contract HIV through vaginal intercourse than men.^8^ This physiological risk is compounded by social risk factors that affect women at higher rates, particularly gender-based violence and rape.^9^

In this paper, we examine how individuals living in 21 high HIV-burden communities invoke caricatures of “woman” when engaging with ideas of “HIV risk”. We explore how people living in these contexts describe women and HIV risk, how HIV is integrated into notions of “womanhood,” and how constructions of “womanhood” shift in social narratives. We ground these discussions about women and HIV in lived social contexts in Zambia and South Africa. These understandings have important health implications for developing interventions and the accompanying HIV-messaging. We argue that local (mis)interpretations of women’s increased HIV risk can sometimes reinforce underlying narratives about women’s inferiority by casting women as lacking the biological and social “wherewithal” to withstand HIV. In contrast, some community narratives explicitly contest such perspectives by highlighting how ongoing gender imbalances in division of labor, care, access, and power perpetuate these epidemiological “vulnerabilities,” and must therefore be shifted. In this way, community narratives about women and HIV risk are complex negotiations with broad implications for the experiences of PLHIV and people living in the context of HIV.

## Background

The portrayal of women as “vulnerable” to HIV is located in both the biological and social risks of HIV acquisition that many women experience. Firstly, women are physiologically at greater risk of contracting HIV per unprotected vaginal sex act than men.^8^ Compounding the increased biological odds is social disempowerment, marginalization, and social risk factors (including risk of violence and assault) that many women face. This disempowerment is illustrated in the UNAIDS Gap Report^10^ where adolescent girls and young women are identified as a vulnerable group in part due to the extent of violence against women. For many women in southern Africa, intimate partner violence has become part of their daily realities.^9^

Secondly, and more broadly, social contexts that reflect wider societal gender relations and hierarchical gendered power dynamics^11^,^12^ can limit women’s agency and indirectly put them at increased risk of contracting HIV. These contextual factors include the often subordinate or dependent economic status of women which is often affected by men’s asserted control over specific sexual domains.^13^ Women also often carry disproportionate responsibility for family care, including care of family members affected by HIV, sometimes to the exclusion of their own health priorities.^14^

More distal risks associated with women’s social contexts include sex work,^15^,^16^ informal transactional sex,^15^,^17^–^19^ or survival sex,^20^,^21^ and alcohol and other substance abuse.^22^ In addition, women may lack power in sexual negotiations, particularly around issues like negotiating condom use.^23^,^24^ Women also lack control over the sexual lives of their partners outside of their relationships, sometimes even when they are aware of their partner’s infidelity.^18^ It should be noted that the notion that women bear the bigger burden of HIV in sub-Saharan Africa is not uncontested.^25^ However, most researchers argue that the biological and social limitations felt by women in sub-Saharan Africa continue to place women at risk. Therefore, women remain a fundamental concern in terms of HIV prevention, treatment, and care.

Researchers have highlighted that when women’s position as subordinate in society is accepted as “normal,” women are often less able to negotiate around health, including negotiating condom use, and are at increased risk of gender-based violence.^26^ While there are often clear social limitations placed on women, the resilience they show should not be ignored.

When taking these varied experiences of vulnerability and resilience into account, Jewkes and Morrell^27^ have argued that femininity is not a singular construct, but rather that there are “multiple femininities” at play in social contexts. As with masculinity,^11^,^12^ there is a hierarchy of femininities. Some femininities (referred to as emphasized femininities^28^) are compliant to patriarchy and the gendered hierarchy, while others are subordinate. Accordingly, while some women are contextually vulnerable to HIV infection and violence, in many other instances women can display significant agency in relationships with men.^27^,^29^ In health literature, however, there has been a tendency to treat women as a homogenous group who are portrayed as victims. Jewkes and Morrell go on to argue that when we use the category of “woman” in an uncritical manner, it diminishes the impact of underlying factors such as women’s needs and desires and how they go about achieving them. In turn, these underlying factors potentially influence women’s relationships with men and their exposure to HIV risk.^27^

### Women as a priority group for HIV programs

Given the uneven distribution of HIV prevalence and risk, women have often been prioritized in prevention and treatment initiatives in southern Africa.^30^,^31^ Many HIV prevention campaigns have focused particularly on the vulnerabilities of women. For example, programs have focused on HIV prevention specifically among female sex workers^32^ on female-controlled prevention technologies (as in the MIRA trial^33^ in Zimbabwe and South Africa), and reducing rates of gender-based violence (as in the Stepping Stones intervention^34^). Programs have also focused specifically on financial or schooling support initiatives for women as a means of empowerment more broadly to negotiate HIV risk.^14^

Women have often been targeted in prevention and treatment interventions because of their roles as mothers and/or wives. For example, Prevention of Mother to Child Transmission (PMTCT) programs are focused on prescribing antiretroviral therapy to expectant mothers to avoid vertical transmission to unborn babies.^35^ In more recent years, PMTCT programs have expanded to retain women on treatment beyond the birth of their babies.^36^

While a large number of programs for the prevention and treatment of HIV are geared toward women, many have met with limited success.^15^ Klein et al^37^ have identified some early structural barriers that might contribute to the varied success of these targeted initiatives. These include economic underdevelopment, poverty, high rates of mobility and migration, social and political instability, and gender inequalities. Despite an acknowledgment of the importance of structural barriers to HIV prevention among women, few interventions directly address gender-related challenges on the ground, often simply positioning women as “vulnerable,” without further exploring implications for effective HIV prevention or treatment interventions. Watts and Seeley have noted that addressing gender inequalities, including gender-based violence, is “not a luxury but a necessity for effective programming, especially in settings where there is a high HIV prevalence among women”.^38^

### Narratives of risk: dialogical self-theory and positionality

To understand the complexity of community narratives about women, HIV risk and notions of vulnerability, we make use of the dialogical self-theory. In dialogical self-theory, the self is defined as a conversation (dialogue) between different voiced positions.^39^ Voiced positions (including experiences of gender) are influenced by various aspects – both internal and shared with others.^40^ In this article, these “voices” represent differing views, assumptions, and positions relative to notions of “woman” and “HIV risk” and thus can offer a lens for explicating the complexity of participants’ perspectives and experiences. The various “voices” are evident in the discourse of participants. van Dijk^40^ noted that discourse is influenced by both “personal and social cognition” which includes “personal memories, knowledge and opinions … [and] those shared with members of the group or culture”. Through voicing these positions people “ascribe rights and claim them for [them]selves and place duties on others”. This positioning of others implies a person or group being located as “trusted” or “distrusted,” “with us” or “against us”^41^ or, in this case, to be “at risk,” “not at risk,” “vulnerable,” “good,” or “bad.” Dialogical self-theory is useful in understanding how community members locate health risks relative to gender as it assists not only in identifying how risk is described, but also identifies the underlying processes behind the allocation of HIV risk.

Our objective is to describe the voiced positions of community members and how they understand HIV risk in relation to the concept of “woman”.

## Methods and analysis

The findings in this paper are drawn from “Broad Brush Survey” (BBS) research conducted with community members in 21 urban, high HIV prevalence communities (for the HPTN 071 [PopART] trial, communities [or trial clusters] were defined as the catchment area of a selected health care center or clinic^42^) in South Africa and Zambia enrolled into the HIV Prevention Trials Network (HPTN) 071 (PopART) study. The BBS entailed a rapid assessment of communities identified to partake in health interventions. The aim of the BBS was to identify key stakeholders, describe the physical layout of the community, identify health services available, and assess community attitudes toward and knowledge of HIV testing, prevention, and treatment. Research activities included conducting a set of group discussions with community members, individual interviews with key informants, and structured observations in communities and health facilities over a period of 12 days (Bond V et al, PopART Broad Brush Surveys [BBS] Technical report for 3ie: Broad Brush Surveys of HIV Prevention, Treatment and Care in 21 Zambian and South African Communities to prepare for HPTN 071 [PopART], unpublished report, 2013). The approach is explained in detail by Murray et al^43^ and Bond et al.^44^ This research methodology is based on participatory methods as used by Wallman et al^45^ and entails rapid data collection to characterize local community context and visible features for bounded communities across descriptive dimensons.^44^ The BBS research was conducted in 21 communities in 2012–2013, prior to the implementation of a community randomized trial of combination HIV prevention (HPTN 071 [PopART]).^42^ In Zambia, communities are distributed across four provinces while the South African communities are all in the Western Cape province. For reporting purposes and to ensure anonymity, research clusters are numbered Z1–Z12 in Zambia and SA13–SA21 in South Africa.

Ethical clearance for the research was received from the University of Stellenbosch Health Research Ethics Committee, the Humanities and Social Sciences Research Ethics Committee at the University of Zambia, and the London School of Hygiene and Tropical Medicine. Permission to conduct the study in health facilities was also granted by the City of Cape Town and the Cape Winelands Provincial Health Research Committee in South Africa and the Ministry of Health and the District Medical Offices in Zambia. Written informed consent was received from all individuals prior to participation in group discussions. Personal identifiers were removed from transcripts prior to analysis and pseudonyms are used throughout this article.

### Study participants

A total of 604 community members (342 of them women) participated in 78 group discussions across the 21 sites in South Africa and Zambia.^44^ Participants were recruited during a process of systematic community observations and were purposively sampled to represent a wide variety of community views (including age and gender). Participants were recruited into groups consisting of “older women” (aged ≥35), “younger women” (aged 18–35), “older men” (aged ≥35), and “younger men” (aged 18–35). Eligibility criteria included living in the study community and being older than age 18. Discussions were facilitated by a graduate-level researcher and a research assistant in the participants’ preferred language (often a mix of English and other local languages).

### Data collection

During the BBS process, semi-structured observations, interviews with key informants, and group discussions with various community members were conducted. The analysis presented here is drawn primarily from one activity conducted in the group discussions with supporting contextualization from other data.

**Figure 1:**
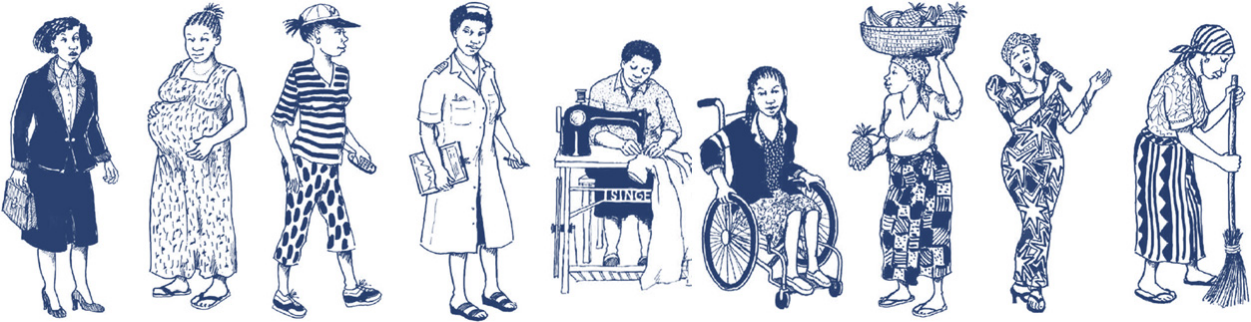
Character cards depicting women used for wealth, poverty, and risk ranking activity. **Notes:** Figures reproduced from Kidd R, Clay S, Chiiya C, editors. *Understanding and challenging HIV stigma: Toolkit for action*. Academy for Educational Development, International Center for Research on Women, and International HIV/AIDS Alliance; 2007. © Illustrations: Petra Röhr-Rouendaal, 2006.^50^

The group discussions were structured around various activities, including a “wealth, poverty, and risk ranking” activity. During this activity, participants were given an assortment of 16 character cards (nine of which depicted women) and asked to select cards that represent “the type of people that live in this community” (Figure 1). The images on the character cards were developed as part of a regional anti-stigma education program implemented in Zambia (images developed by Petra Röhr-Rouendaal on behalf of the Academy for Educational Development, International Center for Research on Women and International HIV/AIDS Alliance). Participants were asked to write down their selected character’s “story,” including descriptions of their income sources, living conditions, families, and health profile in terms of HIV. They then presented the stories to the other participants in the group for discussion.

### Data analysis

All discussions were recorded, transcribed verbatim, quality checked, and translated by trained bilingual researchers into English. Photos of the different character cards and descriptions as well as the transcripts were uploaded into ATLAS.ti (version 7.5.10; Scientific Software Development, Berlin, Germany) for analysis by graduate researchers.

The first phase of analysis entailed identifying all textual extracts where “woman” and “risk of HIV” are mentioned in the “wealth, poverty, and risk ranking” activities. Three of the extracts were independently and inductively coded by the first and second authors^46^ to establish research themes. Coding discrepancies were reconciled and further coding concluded. Three broad categories of associations between “woman” and “HIV risk” were identified – “good,” “bad,” and “vulnerable.” A narrative analysis was then used to identify conversational processes through which each of these three categories of “woman and HIV risk” emerged.

## Findings

Group discussions were held with 158 “older women” (aged ≥35), 166 “younger women” (aged 18–35), 94 “older men” (aged ≥35), and 156 younger men (aged 18–35) (the exact ages of 30 participants [12 men and 18 women] who participated in a “mixed” group discussion are unknown but it was established that they were ≥18 years). We identified three dominant, though not universal, associations between women and HIV risk, which reference archetypal caricatures – “goodness,” “badness,” and “vulnerability.” These characterizations of HIV, women, and risk are illustrative of the narratives of community members and relate to the larger processes related to understanding risk and vulnerability. Further, we found that some participants would shift between these caricatures in describing their perceptions of risk, with the same participant attaching perceived HIV risk to different characteristics at different moments. This finding challenges static explanations of risk, which often assume risk to be consistently attributable to specific characteristics.

We suggest that these shifts in HIV risk perception were premised on three processes: 1) acknowledging the multiple roles women may play; 2) internalizing/personalizing stories about women to fit with lived experiences, and 3) shifting registers in descriptions from what is versus what should be. The archetypal categories of “good,” “bad,” and “vulnerable” are described in the “The good woman, exempt from HIV risk,” “The bad woman, who spreads HIV,” and “The vulnerable woman, at risk of HIV although no fault of their own” sections, respectively.

### The good woman, exempt from HIV risk

In most group discussions, “good” women, as a theme, were defined by participants by a set of behaviors, some of which are linked to dominant public health narratives of HIV prevention. In particular, it was associated with the “abstinence” and “being faithful” components of HIV prevention messaging. Other characteristics extend beyond biomedical HIV prevention methods and include community involvement, attendance at religious institutions (mostly Christian churches), ability to work through hardship, and willingness to take care of families. The following description by one participant illustrates this caricature about a character card depicting a woman singing and wearing a dress and headscarf:

She is a member of the church so she’s obviously working in the congregation of the church … she is helping people every day [when] they have problems … her family is also very very religious … they go to church and I feel because of their lifestyle, they have a very high standard of living [moral standard] and as far as the HIV … there is no risk for them. They work in the community … they [are] cooking food for the poor and they feed the hungry. [Older woman from South Africa, site SA18, December 21, 2012]

### The bad woman, who spreads HIV

The caricatures of “bad” women focus on “risky” (mostly sexual) behavior or putting others at HIV risk. These characterizations often include overtones of moral judgement. This is evident in the following description of a card depicting a female nurse:

Most of the times why the nurses are at a risk is because most of them do not get married. They are just single women … most of the times they do not have husbands so they have a lot of man friends … they can even snatch [someone’s] husband … and start having sex with him. [Older woman from Zambia, site Z2, February 8, 2013]

### The vulnerable woman, at risk of HIV although no fault of their own

The third caricature emphasizes that women are at risk because of circumstances beyond their control. Vulnerability is perceived to exist because of physical threats (rape, abusive partners, violence, and physical disability), social threats (economic vulnerability, survival sex), or circumstantial threats (needle prick injuries, accidents). The attitude toward these women is one of sympathy and commiseration.

There are those lame people (sic). You find that at the house where she stays, there are boys … maybe sometimes she remains alone, they will attack her and take her … remove her from the wheelchair. They go and rape her. When he is done he brings her back and tells her not to tell, she will not tell, she will be quiet. [Older woman from Zambia, site Z10, March 24, 2013)

### Shifting narratives of risk

The three caricatures serve as hypothetical extremes in the narratives of “woman” and “HIV risk” presented by participants. In reality, we argue that, for community members, the complexity and multiplicity of women’s lived experiences are an ongoing, contesting shifting of position between these extremes. The shifting of community members’ understanding happened as different elements of women’s experiences were in the foreground of the narrative. The shifting positioning of “woman” and “HIV risk” could, at first glance, appear contradictory or incoherent. However, participants articulated the complexity of women’s positioning in relation to HIV risk through three rational processes that maintain narrative coherence.

#### Referencing the multiple roles women play in the community

During group discussions, participants (men and women) acknowledged that women fulfil multiple roles in communities, some of which might be considered good, bad, or vulnerable, depending on either the circumstances, the audience, or the position of the woman. The way a woman’s association with HIV risk is described could therefore change depending on the role she was playing. For example, during a group discussion with young men in Zambia, a participant noted the following about a character identified by the group as a sex worker:

For this woman to dress like this, then she doesn’t love herself because a respectable woman is supposed to dress … in a wrapper [skirt]. [Young man from Zambia, site Z7, March 19, 2013]

With this image of the sex worker as the improper, bad woman established by the group, another participant added the following:

You find that they are prostitutes (sic), but they have children at home. They say, ‘I am going for work’, they are just sex workers. Early in the morning they come back home [and] they even buy some bread and small parcels for their children, they have good families, despite that they are sex workers. [Young man from Zambia, site Z7, March 19, 2013]

Through this description there is an acknowledgment that one woman plays multiple roles and the overlapping categories of “good” and “bad” are logically cohesive.

The role of a woman as mother, provider, and wife is also discussed in the following extract from a group discussion with older women in South Africa:

This lady is “Sarah”. She wakes up … to prepare the children to go to school. After she has finished, she cleans the house … She is married and has four kids … She’s [at] risk of getting HIV because her husband … is going to the shebeens [taverns] … he can meet some drinking girls there and then he can end up in bed with those drinkers because he’s also drunk. [He goes] straight to his wife, as dirty as he is … and then the woman [the wife] can get HIV. [Older woman from South Africa, site SA17, March 26, 2013]

In her role as mother, income provider, and homemaker, her character is described as a “good” woman, capable of earning a living and taking care of her children and her home. However, her other role as wife also places her in the perceived position of “vulnerability”.

#### Internalizing/personalizing stories of women

Related to the referencing of multiple social roles that women play, a second process enabled participants to maintain superficially inconsistent caricatures in descriptions of women. Participants’ descriptions of “woman and HIV risk” often blurred the distinction between “a woman” (any woman in their community) and “a particular woman” (such as their mother, sister, or wife). Perceptions and assumptions about “woman and HIV risk” would shift dramatically as the participant changed the referent of their story from “a woman” to “my wife” or “me as a woman” (or vice versa). This blurring between caricatures implies that when the personal history or lived experience of the participant is taken into account, the character is perceived, judged, and presented differently by the participant. In many instances, a community member’s narrative would start as a description of an archetypal character, but during the course of the narrative, personal, internalized experiences would transform the description of the character card. For example, one participant named a pregnant woman depicted on a card “Santie” and described her initially as living in adverse conditions and worthy of sympathy:

She looks like a person who is very poor, [she] seems like [someone] who is raising her child on her own, that doesn’t have parents and she, for me it would seem like, I can say, she only visits her friends … that is almost like for her family, because she looks like she no longer has family … it makes me feel bad now … The person seems to me like someone who is suffering, you see. ‘Santie’ seems to me like someone who suffers, that doesn’t work … she has HIV, uhm, HIV/AIDS. [Young woman from South Africa, site SA21, April 10, 2013]

In this case, the description of “Santie” begins as referencing the vulnerability caricature. However, as the narrative develops there is a shift from the sympathetic description of “Santie” to also acknowledging the general “bad” behavior of women witnessed in the community.

There are people taking care of her baby for the hard times … here in SA21 it happens like that, hey … they make children then leave them with you, just like that. [Young woman from South Africa, site SA21, April 10, 2013]

In this discussion, one participant – a young woman herself – described other young women in her community as actively engaging in sex (“making children”) and leaving their children (“just like that”) in harder times. Following this general description of women in the community, the same participant shifts her description toward blaming/vilifying “Santie” for not testing for HIV during pregnancy and passing on the virus to her child:

She now has the AIDS and … in the end it will be passed onto the baby now … perhaps if she went for her test and she found out … then she [would have] realised she [has] AIDS … As I can see [laughing] she looks like a person who was sleeping around and doesn’t know who the child’s father is. [Young woman from South Africa, site SA21, April 10, 2013]

In this instance, the shift from “vulnerable” to “bad” is gradual, but purposeful. The participant started with a clear, sympathetic narrative of “Santie,” but soon acknowledged her own experiences of women in her community, and fit “Santie” into her experience of young women as “bad” women.

#### What is versus what should be

Associations between women and HIV risk also shifted markedly along these continuums when participants shifted register between what their understandings of women are or what they understood constituted HIV risk is and what it should be. In the following example, a young man from Zambia initially describes women in the community through the broad lens of expected “badness”:

School girls of nowadays, they do not date their sizes [age group]. They will go for those sugar daddies … every day, every weekend they are out with the teacher or something … even if someone their age tried to propose them they will say, ‘What will this one offer me?’ … They are just after money … they date men like their parents’ age, those elderly men, working class who are stable and married … These school girls are at very high risk of HIV, like in the compounds. [Young man from Zambia, site Z11, January 22, 2013]

However, immediately after this description of the “bad” young women, there is a shift in register from the participant to acknowledge the survival pressures that young women experience:

You find that where the young girl is coming from things are not good, life is hard. In the end she just thinks, maybe this one will keep me well. [Young man from Zambia, site Z11, January 22, 2013]

In his narrative, there is a self-awareness of the mismatch between moral expectation and circumstantial pressure, which is accounted for by the shift in register. This is also evident in the following example, in which another young man describes a character card depicting a pregnant woman:

[Zola] has devoted [herself] to be a housewife; she will clean, so that her house is clean … she’s not working. She has children. [Young man from South Africa, site SA14, June 3, 2013]

When asked about Zola’s risk of HIV, the participant answered:

On HIV, if I sleep around with other people, you see, I sleep with other people that I don’t know … even how they look, you won’t see how the person is. Now you just see the person’s beauty, you’ll see the beauty and sleep with them without a condom. [Young man from South Africa, site SA14, June 3, 2013]

The participant’s narrative changes the description of the “good,” devoted wife to someone who is potentially the one who passes on HIV to others, including himself. He notes that risks are taken with beautiful strangers and acknowledges that there is more to HIV risk than what could be seen on the surface.

A superficial analysis of participant narratives could point to quite stark, unsophisticated caricatures of women as either “good,” “bad,” or “vulnerable.” However, it is through paying attention to the shifting between such extremes that the complexity of community narratives about women and HIV risk can be explicated. The blurry, contested nature of associations between women and HIV risk is illustrative of the multifaceted, difficult, and tenuous positions that women living in high HIV prevalence contexts occupy.

## Discussion

The aim of our research was to characterize the diversity of narratives that are thematically relevant to critically understanding the ways in which people speak about women and HIV risk in looking for patterns in narrative. In this, we found that community members were aware of the ways in which women challenged the positions that they were believed to assume (“good”, “bad”, “vulnerable”) and were able to articulate how HIV risk is a shifting phenomenon.

When shifting through categories of risk, community members often invoked the multiple roles that women play in society. In doing so, they referenced broader gendered power dynamics in society. Further, in their changing descriptions of women and the positions that they could assume, community members in our study either reinforced dominant femininities (for example the “good woman” who completes domestic duties and takes care of her children and husband) or challenged these femininities (where the “bad woman,” the sex worker, is also able to provide for her children). Their descriptions of multiple femininities highlight how women can be seen not only to employ different roles, but also to exist in multiple stages of moving between being at risk/not at risk of contracting HIV.

In describing the multiple but simultaneous stages of risk, the concept of dividuality proves useful. Helle-Valle^47^ defined dividuality as potential different perspectives that individuals may employ depending on the social context that they find themselves in. The findings demonstrate how individuals shift their perceptions of risk not only based on their understanding of HIV, but also on the context in which they live. The changing context also links to theories of intersectionality, as described by Crenshaw,^48^ where the multiple spaces of intersecting vulnerability (such as race and gender) that women occupy are acknowledged. These theories engage with multiple perspectives and viewpoints that individuals can rationally employ at any given point in time.

In addition to addressing broader understandings of the position of women and concepts of risk, our findings also offer important insights for the implementation of public health interventions related to HIV, which must address gender dynamics and power inequalities in order to be effective. Public health prevention and treatment messaging have often focused on women’s vulnerability or on their need to avoid “bad” behavior (as in abstinence messaging).

However, our analysis shows that there are positive versions of women that could potentially be drawn on when considering HIV prevention and treatment messaging. The analysis highlights that women are diverse and that many of the positions they occupy are positive. This is also evident in the story of Zola, as told by a young man in South Africa. Zola, although described to be at risk of HIV because of the behavior of her husband, is also seen as a healthy, contributing, member of society. Zola is employed and is the caregiver of her children.

In addition, regardless of how a woman (or person) is cast, this perception can change over time. The analysis shows that community members’ perceptions of women are not stagnant in their positions of potential HIV risk, but rather that there is a “shift-ability”. This can be seen in the story described by the young man in Zambia of the sex worker (engaging in “bad” behavior) who is also a responsible mother. Messaging that relates only to one aspect of her life (her position as sex worker) and ignores the other (her position as mother) risks stigmatizing certain behaviors and, by extension, certain groups. Health implementers need to consider health messaging beyond the simple casting of a character (or person) into a fixed category of risk.

Lastly, while public health researchers and practitioners categorize risk in certain key populations (defined by WHO^49^ as population groups disproportionately affected by HIV; usually limited to men who have sex with men, transgender people, sex workers, and intravenous drug users), we suggest that all women, including those who are not considered part of specific key populations, should also be considered as a special interest group in public health efforts to address the HIV epidemic. This includes broadening the focus of what vulnerability entails, and focusing on positive messaging as well.

## Study limitations

Participants were recruited from public spaces (at the clinic, library, public transport hubs, and shopping areas) and there is a likelihood that community members who do not frequent these spaces are under-represented and should be further researched. However, through the analysis, we included representation of a variety of group discussions from various communities across countries to ensure that we were able to represent the processes at play in understanding women and HIV risk.

Our analysis shows the processes through which community members in these study communities understand HIV risk. We have not proven that these processes are exhaustive, universal, or equally applicable in all contexts. Instead, they provide a useful starting point for considering how to escape narrow caricatures of associations between women and HIV risk. The illustrative quotations presented here are thus purposively selected to explicate the diversity and complexity of perceptions about women and HIV risk in real-world talk.

## Conclusion

In summary, our analysis has shown that, in consultation with affected communities, a wider understanding of the lived experiences of women and the various potential stages of being at risk/not being at risk of HIV could be beneficial for the implementation of efficient HIV prevention programs.

Our participants described the multiple positions that women occupy not as contradictory, but rather as an accurate reflection of the multifaceted realities of women in communities with high HIV prevalence. Further, this paper has shown that community members’ perception of being “at risk” is not a static position and that women, and community understandings of women, go through multiple shifts.

Our analysis has implications for understanding how perceptions of HIV risk can influence the perceived and real position of women and how these positions shift. Interventions that target specific groups of at-risk women will not be sufficient to reduce women’s risk of HIV acquisition, as shown in the intervention studies focusing only on sex workers, female-controlled prevention technologies, gender-based violence, or financial and schooling support.^15^,^32^ Focusing only on the contexts where women are perceived to be most at risk could potentially mean missing out on women who might be at risk at a different time. Instead, health implementers would benefit from taking into consideration the multiple positions women employ in different contexts (including positions of power, or positions perceived to be devoid of risk) as well as how the wider community’s understandings of women and “risk” might shift when designing and implementing HIV prevention and care programs. This can be achieved through more active consultation with various local community groups with discussions that extend beyond simply defining HIV risk but aim to explore the everyday roles and realities of men and women.
